# Metal Ionophore Treatment Restores Dendritic Spine Density and Synaptic Protein Levels in a Mouse Model of Alzheimer's Disease

**DOI:** 10.1371/journal.pone.0017669

**Published:** 2011-03-11

**Authors:** Paul A. Adlard, Laura Bica, Anthony R. White, Milawaty Nurjono, Gulay Filiz, Peter J. Crouch, Paul S. Donnelly, Roberto Cappai, David I. Finkelstein, Ashley I. Bush

**Affiliations:** 1 Oxidation Biology Laboratory, The Mental Health Research Institute, Parkville, Victoria, Australia; 2 Synaptic Neurobiology Laboratory, The Mental Health Research Institute, Parkville, Victoria, Australia; 3 Department of Pathology, The University of Melbourne, Parkville, Victoria, Australia; 4 Centre for Neuroscience, The University of Melbourne, Parkville, Victoria, Australia; 5 Bio21 Molecular Science and Biotechnology Institute, The University of Melbourne, Parkville, Victoria, Australia; 6 School of Chemistry, The University of Melbourne, Parkville, Victoria, Australia; Nathan Kline Institute and New York University School of Medicine, United States of America

## Abstract

We have previously demonstrated that brief treatment of APP transgenic mice with metal ionophores (PBT2, Prana Biotechnology) rapidly and markedly improves learning and memory. To understand the potential mechanisms of action underlying this phenomenon we examined hippocampal dendritic spine density, and the levels of key proteins involved in learning and memory, in young (4 months) and old (14 months) female Tg2576 mice following brief (11 days) oral treatment with PBT2 (30 mg/kg/d). Transgenic mice exhibited deficits in spine density compared to littermate controls that were significantly rescued by PBT2 treatment in both the young (+17%, p<0.001) and old (+32%, p<0.001) animals. There was no effect of PBT2 on spine density in the control animals. In the transgenic animals, PBT2 treatment also resulted in significant increases in brain levels of CamKII (+57%, p = 0.005), spinophilin (+37%, p = 0.04), NMDAR1A (+126%, p = 0.02), NMDAR2A (+70%, p = 0.05), pro-BDNF (+19%, p = 0.02) and BDNF (+19%, p = 0.04). While PBT2-treatment did not significantly alter neurite-length in vivo, it did increase neurite outgrowth (+200%, p = 0.006) in cultured cells, and this was abolished by co-incubation with the transition metal chelator, diamsar. These data suggest that PBT2 may affect multiple aspects of snaptic health/efficacy. In Alzheimer's disease therefore, PBT2 may restore the uptake of physiological metal ions trapped within extracellular β-amyloid aggregates that then induce biochemical and anatomical changes to improve cognitive function.

## Introduction

Alzheimer's disease (AD) is characterized by deficits in higher order cognitive processes. One of the principal substrates for this dysfunction is spine/synaptic loss, which studies in AD animal models have demonstrated is most pronounced in the immediate vicinity of Aβ plaques [1], [2], [3]. Furthermore, Aβ oligomers impair NMDAR-dependent signalling cascades, ultimately resulting in the progressive loss of dendritic spines and glutamatergic synapses [4], [5], [6].

One of the mechanisms likely to mediate such deleterious effects is perturbed trafficking of essential synaptic metal ions, such as zinc. Synaptic Zn^2+^ functions as a neuronal messenger and a modulator of synaptic transmission and plasticity through targeted interactions with proteins such as TrkB and NMDAR2b [7], [8]. The attraction of Aβ oligomers to Zn^2+^ emanating from the glutamatergic synapse selectively occludes the NMDAR2b subunit [9]. Thus, the sequestration of Zn^2+^ in oligomeric Aβ-Zn complexes may lead to a reduction in zinc turnover at the synapse, limiting the trans-synaptic movement of zinc to modulate post-synaptic targets, and resulting in impaired cognition.

Drug candidates clioquinol (CQ) and PBT2 (Prana Biotechnology Ltd) have a moderate affinity for metal ions, and rather than deplete biological metals in cell culture, promote the uptake of Cu and Zn [10]. We have previously demonstrated [10] that brief administration of CQ and PBT2 (11 days, 30 mg/kg) to both Tg2576 and APP/PS1 transgenic mice resulted in decreased brain Aβ burden with rapid (within 5 days) and marked improvements in learning and memory performance on the Morris water maze. PBT2 also induced cognitive benefits in a phase IIa clinical trial [11], [12]. To understand the mechanisms that underlie this restoration of cognition, we studied the effect of PBT2 on synaptic plasticity-related endpoints in both cell culture and Tg2576 mice.

## Materials and Methods

All animal procedures were approved by the Howard Florey Institute animal ethics committee (Melbourne, Australia), and were carried out in accordance with the Australian code of practice for the care and use of animals for scientific purposes as described by the National Health and Medcial Research Council of Australia. Female Tg2576 animals were group-housed and aged to 4 or 14 months. For 11 days prior to culling, animals were given a daily oral gavage of sham (standard suspension vehicle [10]) or PBT2 (30 mg/kg, provided by Prana Biotechnology Ltd, Melbourne, Australia) in the same vehicle (n = 8–12 mice/group total; split between golgi and biochemical analyses). One hour following the last drug dose, animals were culled and the brain either immediately processed for Golgi analysis, or frozen at −80°C for subsequent biochemical analyses. The dose of PBT2 was chosen based upon our own historical data, where we have shown that this level of exposure to the compound effectively modulates a number of biochemical and behavioural parameters in various transgenic mouse models of AD, but does not alter bulk brain metal levels [10]. With regards to how this relates to a human dose, based upon FDA guidelines a dose of 30 mg/kg in a mouse equates to a dose of 183 mg/day in a 75 kg man. Our published human clinical trial data demonstrated that PBT2 was found to be therapeutic at 250 mg/day [11], [12].

### Golgi staining

A subset of animals (n = 3–5 mice/group) were deeply anaesthetized with sodium pentobarbitone (100 mg/kg) and then transcardially perfused with 0.01 M cold PBS. Brains were then rapidly removed and cut into 4 mm blocks. The blocks containing the hippocampus were incubated in rapid golgi solutions (Rapid Golgi Stain Kit, MTR scientific), based on the methods of Glasser and Van der Loos [13]. The blocks were infused with a solution containing potassium dichromate, potassium chromate and mercuric chloride for two weeks. The blocks were then snap frozen and cut at 90 µm using a microtome (Cryostat, Leica CM18-50, Leica Microsystems, Nussloch GmbH, Germany). Sections were collected at the level of the hippocampus (bregma −1.40 to −2.70) and thaw-mounted onto gelatinized microscope slides. The slides were dried, dehydrated, cleared with xylene and mounted with distyrene plasticized xylene (DPX). Golgi-treated brain tissue was analysed with a light microscope with an oil immersion lens (63x, NA 1.3; Leica DM4000B, Leica Microsystems; with a further magnification from the projection lens to the camera (10x) used for all measurements, providing a total magnification of 630 times). The whole neuron was traced manually.

Five neurons from each animal (n = 3–5 animals/group) were randomly selected for hippocampal spine analysis. Neurons were selected only if; they were clearly identified as being from the CA1 subfield, the neurons appeared completely filled and they were far enough from the neighbouring Golgi stained cells to be individually identified. Tertiary or greater order apical and basal dendrites (n = 5 apical and n = 5 basal) were selected and analysed to determine spine density, dendritic length and dendritic number (a cartoon outlining this method is shown in Figure S1). Thus, 50 dendrites were sampled from each animal. The standard deviations indicate the uniformity of the analyses and demonstrate that this is a representative sampling.

### Biochemical analyses

Western blot, as previously described [14], was used for the quantitation of protein levels in individual hippocampi taken from a subset of animals (n = 5–7 mice/group). The antibodies utilized are outlined in Table S1.

### Cell culture

Diaminosarcophagine (1,8-diamino-3,6,10,13,16,19 hexaazabicyclo[6.6.6]icosane), abbreviated to (NH_2_)_2_sar and given the trivial name “diamsar”, was synthesised as previously reported [15].

For biochemical studies, SH-SY5Y cells were utilised (in DMEM+10% serum), cells harvested after one hour exposure to the various treatments and then western blots performed (n = 6 replicates for each treatment).

Neurite outgrowth studies, which were conducted separately from the biochemical studies, utilised PC12 cells (grown in DMEM+10% fetal bovine serum) treated with NGF at 50 ng/ml in serum-free DMEM for 48 h. DMSO vehicle, 150 nM PBT2, 150 nM CuCl_2_, 150 nM ZnCl_2_, 150 nM PBT2-Zn (equimolar PBT2 and ZnCl_2_), 5 µM diamsar, 150 nM PBT2 plus 5 µM diamsar or 150 nM PBT2-Zn plus 5 µM diamsar were then added for 24 h. Treated cells were photographed and Image J software (NIH) used to determine the proportion of cells that had neurites 2x or longer than the width of the cell body, and to obtain a mean neurite length (n≥6 images from 3 cultures for each treatment).

### Statistics

All analyses were performed by an operator that was blinded to the experimental conditions. An ANOVA followed by a post-hoc Tukey's multiple comparison test was performed for all comparisons (GraphPad Prism Version 5.0b).

## Results

Initial assessment of 4 month old wildtype animals did not reveal any significant effect of PBT2 on dendritic spine density (apical  = −7.5% and basal  = −1.8%; both compared to sham-treated wildtypes), suggesting that there was no generic effect of PBT2 on this endpoint. This is consistent with our previous report, where there was no effect of PBT2 on the performance of wildtype animals in the Morris water maze [10].

### PBT2 increases hippocampal apical spine density in Tg2576 mice

**Figure 1:**
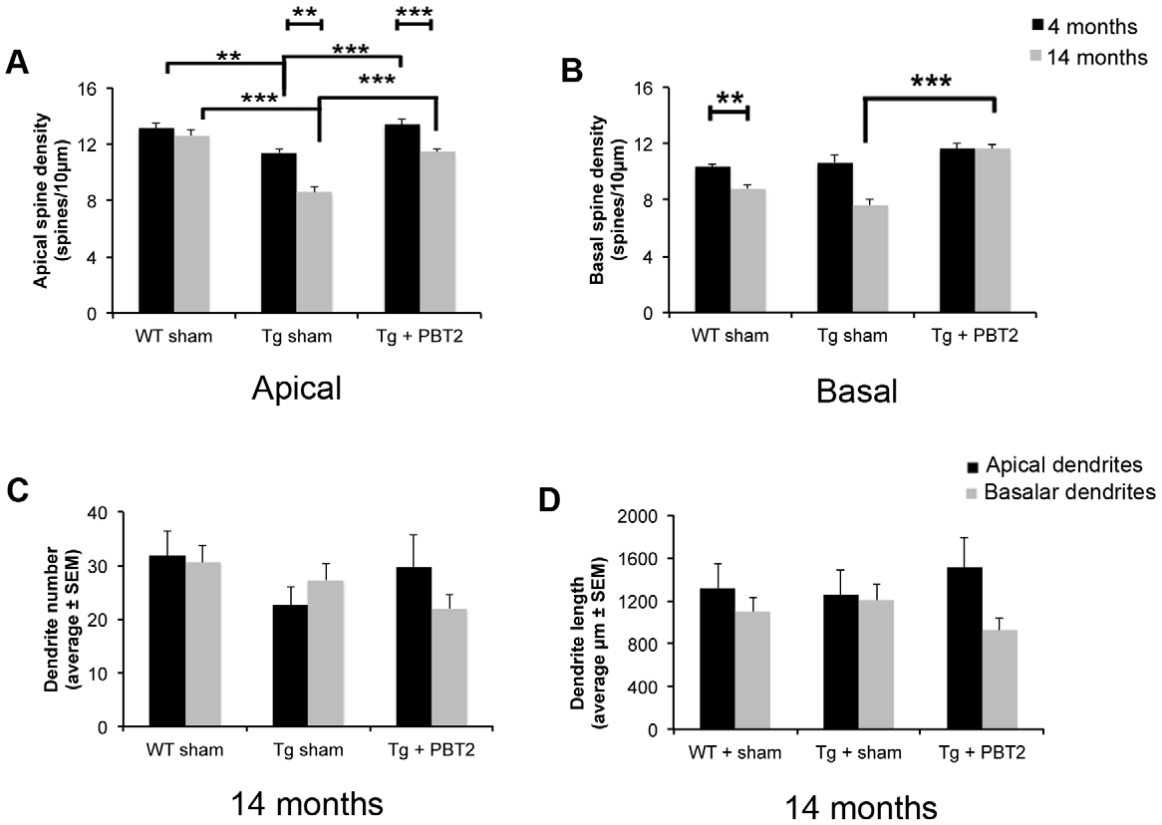
Dendritic spine analysis in Tg2576 mice and wildtype littermates. (A) Tg2576 animals have deficits in apical dendritic spine density at both four (n = 4) and fourteen (n = 5) months of age, as compared to WT mice (n = 4 at four months; n = 5 at fourteen months), that are reversed by short-term (11 days) PBT2-treatment (n = 3 at both age points). There are also age-related decreases in apical spine density in both the Tg groups. (B) There is a significant increase in basal spine density in fourteen month old Tg animals treated with PBT2. There is also an age-related decrease in basal spine density in WT animals. (C) There were no significant differences in apical or basal dendrite number across the experiment. (D) There were no significant differences in apical or basal dendrite length across the experiment. Values are mean ± SEM. **p<0.01, ***p<0.001.

Consistent with previous reports [16], [17], we demonstrated that Tg2576 mice have decreased apical spine density compared to age-matched WTs (sample image in Figure S2). These deficits occurred as early as 4 months of age (−13%, p<0.01) and were more severe at 14 months of age (−31%, p<0.001) (Figure 1A).

Treatment of young Tg2576 mice with PBT2 for 11 days resulted in an increase in apical spine density (+17%, p<0.001) that abolished the deficits seen in younger sham-treated Tg animals (Figure 1A). In older animals, PBT2-treatment also resulted in an increase in apical spine density (+32%, p<0.001), which approached WT levels (Figure 1A).

There was also an age-related decrease in apical spine density evident only in both Tg groups (sham-treated, −24%, p = 0.001; PBT2-treated, −15%, p<0.0001) (Figure 1A).

### PBT2 increases basal spine density in Tg2576 mice

There were no genotype-specific changes in basal spine density at either age (Figure 1B). There was, however, an age-related deficit in basal spine density observed in WT (−15%, p = 0.0017) and a similar trend in the sham-treated Tg mice. In contrast, 14 month old Tg2576 mice treated with PBT2 had greater basal spine density (+26%, p<0.001) than sham-treated controls, with basal spine density reaching similar levels to the 4 month old mice (Figure 1B).

There were no significant changes in dendritic number or length observed in 14 month old animals treated with PBT2 (Figure 1C and 1D).

### PBT2 restores biochemical substrates of learning/memory in Tg2576 mice

**Figure 2:**
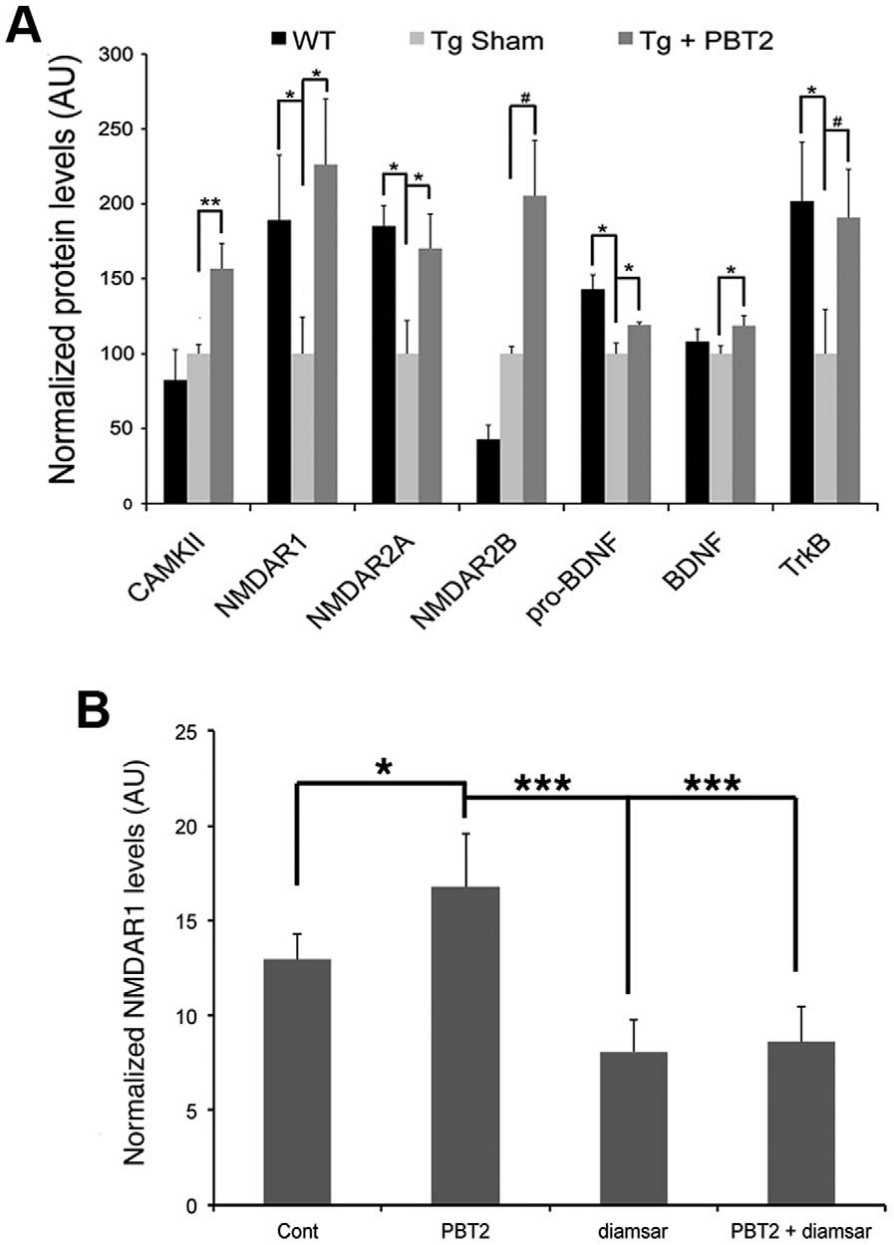
Biochemical profiles in Tg2576 and wildtype littermate mice and in cell culture. (A) Tg2576 mice have significant deficits in a number of proteins, as compared to age-matched wildtype controls, and PBT2-treatment significantly increases the expression of a number of these proteins to the levels seen in the control mice. Values have been normalized to the mean of Tg Sham group = 100. (B) The effect of PBT2 on NMDAR1 protein levels in SH-SY5Y cells is abolished in the presence of the high affinity chelator, diamsar (n = 6/treatment). *p<0.05, **p<0.01, #p = 0.06.

To explore biochemical mechanisms underlying these effects, we assessed the levels of various proteins involved in plasticity (Figure 2A; sample blots shown in Figure S3). Both BDNF and its precursor, pro-BDNF, were increased (+19%, p = 0.04; +19%, p = 0.02, respectively) in PBT2-treated compared to sham-treated Tg2576 mice. The high-affinity receptor for BDNF, TrkB, showed a trend to elevation in PBT2-treated Tg2576 mice (+91%, p = 0.06). In addition, CamKII was elevated (+57%, p = 0.005) following PBT2 treatment.

PBT2 treatment of Tg2576 mice also increased levels of NMDAR1A and NMDAR2A (+126%, p = 0.02; +70%, p = 0.05 respectively), with a trend to an elevation in NMDAR2B (+105%, p = 0.06). As one of the most significantly upregulated proteins we also sought to test the metal-dependence of this effect in vitro. PBT2 treatment resulted in an increase in NMDAR1A protein levels (+29%, p = 0.02) that was abolished by diamsar (−49%, p<0.0001). Diamsar also reduced NMDAR1A protein levels below that found in controls (−34%, p = 0.002) (Figure 2B).

Other proteins examined in the in vivo tissues, including AMPAr, SNAP25, synaptotagmin and PSD95 were not significantly altered, although Creb trended towards an increase following PBT2-treatment (Figure S4).

### PBT2 increases neuritogenesis in a metal-dependent manner in vitro

Although there was no significant effect of PBT2 on dendritic length in vivo, our own qualitative in vitro observations (data not shown) suggested that PBT2 was altering the appearance of cells grown in culture. As it is possible that the treatment period used in our in vivo studies was not sufficient to evince this morphological change, or that there was not sufficiently high local concentrations of drug to see an effect, we sought to examine the trophic effects of PBT2 in vitro and to assess the reliance of any effects on the entry of metals into cells.

**Figure 3:**
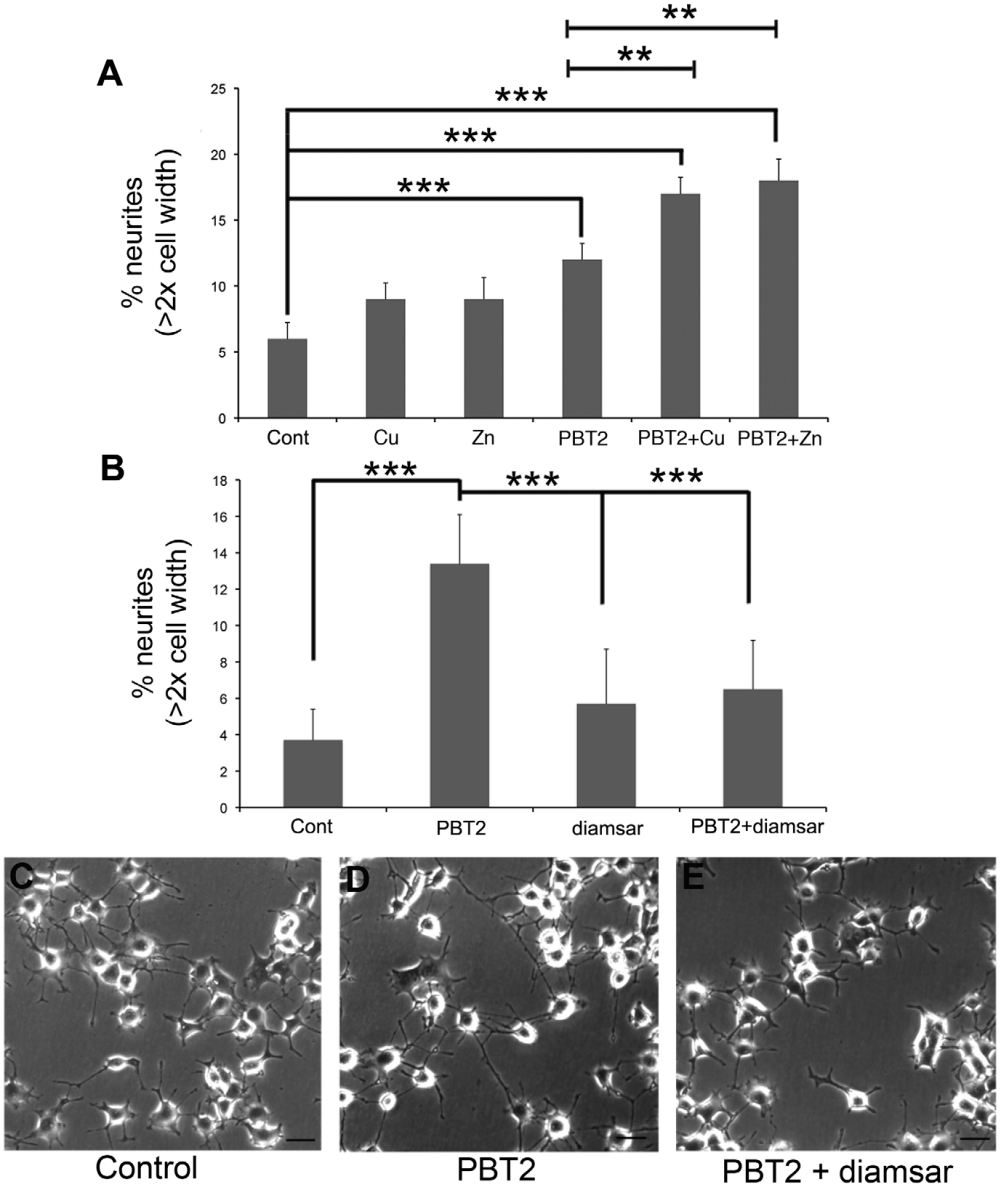
The metal-dependent effect of PBT2 on neurite outgrowth. The effects of copper and zinc alone (0.15 µM) or complexed to PBT2 (0.15 µM) were assessed. (A) PBT2 induces an increase in neurite outgrowth in PC12 cells that is exaggerated when PBT2 is complexed to either copper or zinc (n = 6/treatment). (B) The neuritogenic effect of PBT2 is abolished in the presence of the high affinity chelator, diamsar (n = 9/treatment). Neurite outgrowth in PC12 cells, under control (C), PBT2 (D) and PBT + diamsar (E) conditions is shown in the bottom micrographs (scale bar  = 200 µm). Values are mean ± SEM. **p<0.01, ***p<0.001.

PBT2 treatment resulted in a robust increase in neurite outgrowth (+200%, p = 0.006) (Figure 3A), which we hypothesized was due to complexing with Zn or Cu in the buffer and then increasing cellular metal content via its ionophore activity. Indeed, neurite outgrowth was enhanced when PBT2 was complexed with either Cu or Zn (+300%, p<0.0003) (Figure 3A). Cu and Zn alone, however, did not significantly increase neurite outgrowth (Figure 3A).

Furthermore, PBT2-induced neuritogenesis required cellular metal influx, since it was abolished by co-administration of the high affinity metal chelator, diamsar (Figure 3B–3E). MTT assay demonstrated that none of the treatments impaired viability (+55%, p = 0.04) (Figure S5).

## Discussion

We have demonstrated that brief administration of PBT2 in a transgenic mouse model of AD rectifies deficits in key biochemical memory substrates, which may explain the rapid improvement in cognition seen in these animals during treatment. We also demonstrated that these effects could be mediated by metal ion uptake, consistent with the hypothesized mechanism of action for this class of compound [10].

We have recently demonstrated that the short-term administration of the ionophores CQ and PBT2 to both APP/PS1 and Tg2576 mice significantly reduced Aβ burden and the hyper-phosphorylation of tau [10]. These changes in classical AD biomarkers were accompanied by a concomitant increase in synaptophysin, a surrogate marker for synapses, and a striking restoration of performance on the Morris water maze. The behavioural effects were evident after less than a week of treatment, and over the course of the study the ionophore-treated Tg animals performed as well as or better than wildtype control mice. These benefits were likely to be mediated by biochemical and synaptic benefits that may result from both a reduction in Aβ burden and the mobilization of Zn and Cu trapped in Aβ aggregates.

Consistent with previous reports [16], [17], and actual AD [18], [19], [20], we found that Tg2576 mice have deficits in dendritic spine density (Figure 1). This was observed at 4 months of age and was more pronounced by 14 months of age. PBT2 treatment resulted in a near-complete restoration of apical spine density in both young and old Tg animals, in addition to reversing the age-related decrease in basal spine density in the Tg animals (Figure 1).

Such anatomical changes must be supported by local protein synthesis. We therefore analysed levels of key proteins involved in synaptic function to gauge the impact of PBT2 on plasticity-related events. The most prominent effects of PBT2 treatment were on NMDA receptors (NMDAR1, 2A, 2B), which were increased ∼100% above levels in sham-treated Tg mice (Figure 2). This is consistent with the increase we observed in spine density (Figure 1), which express glutamate receptors (such as NMDAr), whose activation promote spine stability [21], [22], [23]. The downstream signalling events from these glutamate receptors are mediated by a variety of proteins, including CaMKII, which similarly showed a >50% increase following PBT2 treatment (Figure 2). Signalling proteins such as CaMKII, which is upregulated within the post-synaptic density following neuronal activity, modulate glutamate receptor levels and spine morphology [24], [25], [26]. Indeed, the induction of LTP results in an enlargement and proliferation of spines in a NMDAr and CaMKII-dependent manner [27], [28], [29], [30], [31], [32]. Additionally, spine survival is promoted by elements of the BDNF pathway [33]. BDNF, pro-BDNF, and TrkB, were all elevated following PBT2 treatment (Figure 2), consistent with a neurotrophic response.

Thus, PBT2 treatment results in a significant upregulation of key proteins that is likely to result in a more active synapse, increased structural plasticity and enhanced cognitive function. In Tg2576 mice, where many of these pathways are deficient in comparison to WT mice, this may explain the ability of PBT2 to rapidly restore cognitive function.

Our observation that the effects of PBT2 are dependent upon increasing metal uptake is consistent with our recent findings that the expression of several of these proteins are dependent on synaptic zinc content [14]. Aged ZnT3 KO animals, which have a specific absence of synaptic zinc, show significant deficits in proteins such as BDNF, pro-BDNF, TrkB and NMDA receptors. In AD, extracellular amyloid traps Zn^2+^ and Cu^2+^ [34] and may deprive the synapse of these trophic metal ions. Thus, the efficacy of PBT2 in modulating synaptic plasticity, by reversing extracellular metallostasis, may serve as one of its primary mechanisms of action underlying the improved cognition that results following treatment in both mice [10] and humans [11], [12].

## Supporting Information

Figure S1 **Cartoon showing the quantitation of golgi data.** A schematic of the hippocampus, showing the different sub-regions (CA1-3 and dentate gyrus (DG)), is shown in (A). A representative golgi-stained image is then shown in (B), with the traced neuron shown in (C). Also shown in panel (C) is the orientation of the dendrites (basal/apical), together with the cell body (CB) and the branching of the dendrites into the primary (1), secondary (2) and tertiary (3) dendrites. The blue boxes represent the tertiary dendrites from which spine counts were made. Panel (D) shows a representative dendrite with spines (arrow).
(TIF)

Figure S2 **Examples of golgi data.** The left hand panel shows both apical and basal dendrites. The three central figures show typical line-drawings for dendrites from each of the three treatments, where there is no significant change in dendrite length or density. The images on the right hand side show images used for calculating spine density. The Tg animals clearly have a decreased number of spines per neurite, as compared to WT mice, and this is normalised by PBT2 treatment.
(TIF)

Figure S3 **Representative western blots.** (A) shows the various antibodies used in Figure 2A in the main text, while (B) shows a representative blot for the data presented in Figure 2B in the main text. All blots were normalised to GAPDH as a loading control. WT = wildtype; Cont = control.
(TIF)

Figure S4 **Biochemical profile of proteins that were not significantly altered with PBT2 treatment.**
(TIF)

Figure S5 **MTT assay on SH-SY5Y cells treated with PBT2 ± diamsar.** PBT2-Zn treatment results in a significant increase in cell viability. Values are normalised to control = 100% and are means ± SEM *p<0.05.
(TIF)

Table S1 **The various antibodies used for western blot.**
(TIF)
