# Supplementary figures and images for: Metal Ionophore Treatment Restores Dendritic Spine Density and Synaptic Protein Levels in a Mouse Model of Alzheimer's Disease
Source: PLoS One. 2011 Mar 11;6(3):e17669. doi: 10.1371/journal.pone.0017669 (PMC3055881; doi:10.1371/journal.pone.0017669)

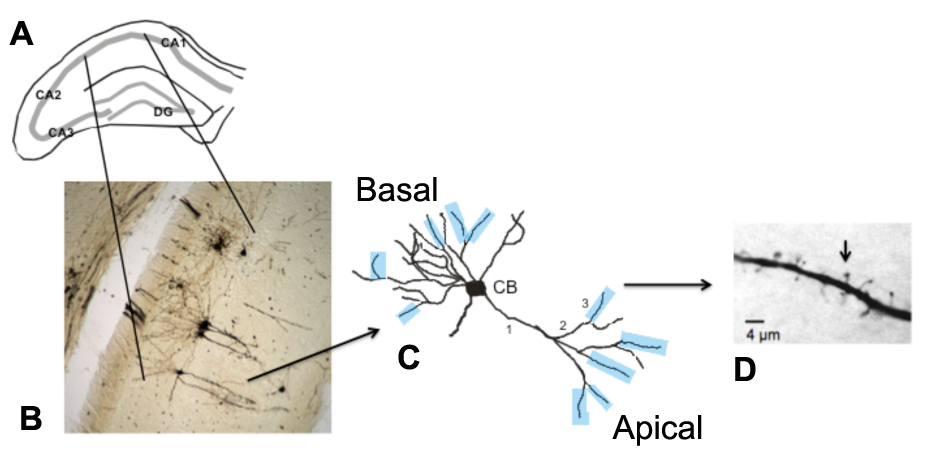

Supplement: Figure S1 — Cartoon showing the quantitation of golgi data. A schematic of the hippocampus, showing the different sub-regions (CA1-3 and dentate gyrus (DG)), is shown in (A). A representative golgi-stained image is then shown in (B), with the traced neuron shown in (C). Also shown in panel (C) is the orientation of the dendrites (basal/apical), together with the cell body (CB) and the branching of the dendrites into the primary (1), secondary (2) and tertiary (3) dendrites. The blue boxes represent the tertiary dendrites from which spine counts were made. Panel (D) shows a representative dendrite with spines (arrow). (TIF) [file pone.0017669.s001.tif]

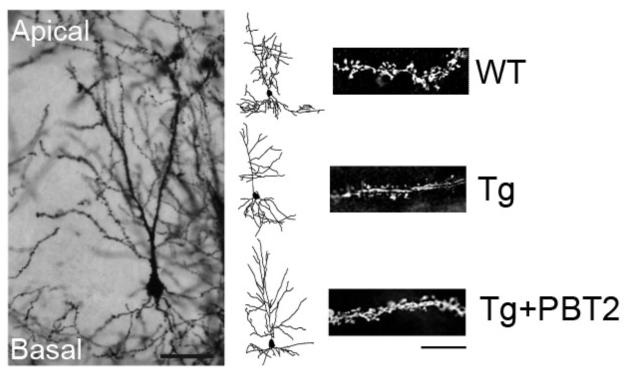

Supplement: Figure S2 — Examples of golgi data. The left hand panel shows both apical and basal dendrites. The three central figures show typical line-drawings for dendrites from each of the three treatments, where there is no significant change in dendrite length or density. The images on the right hand side show images used for calculating spine density. The Tg animals clearly have a decreased number of spines per neurite, as compared to WT mice, and this is normalised by PBT2 treatment. (TIF) [file pone.0017669.s002.tif]

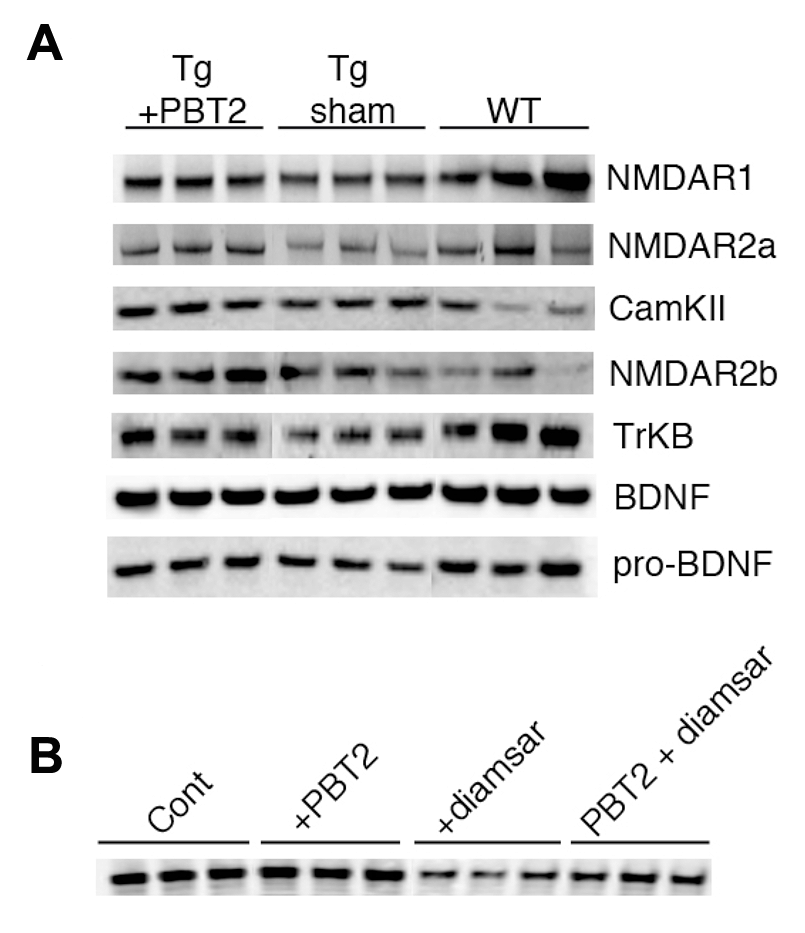

Supplement: Figure S3 — Representative western blots. (A) shows the various antibodies used in Figure 2A in the main text, while (B) shows a representative blot for the data presented in Figure 2B in the main text. All blots were normalised to GAPDH as a loading control. WT = wildtype; Cont = control. (TIF) [file pone.0017669.s003.tif]

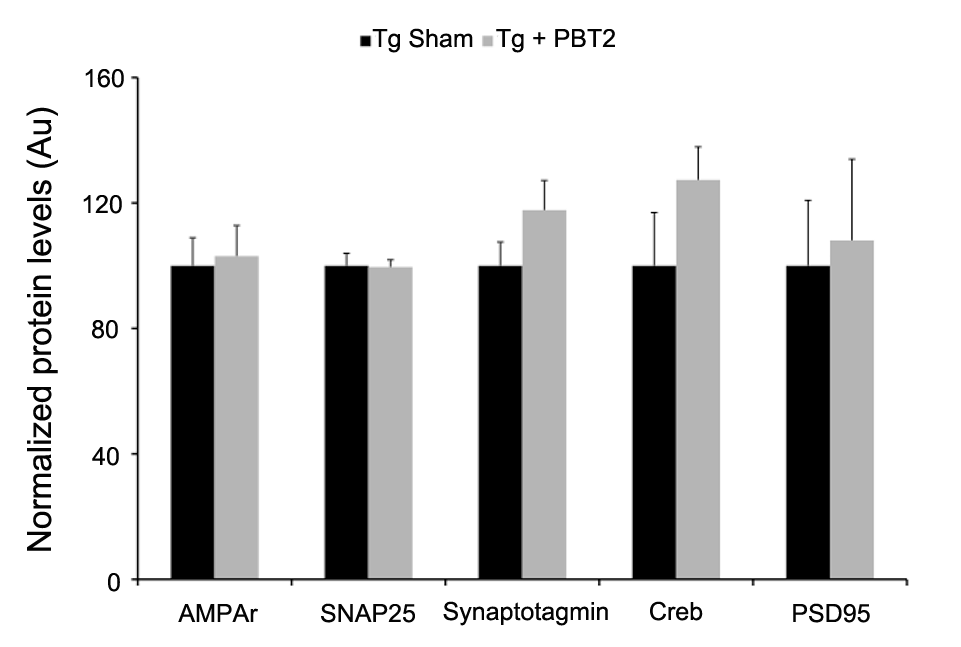

Supplement: Figure S4 — Biochemical profile of proteins that were not significantly altered with PBT2 treatment. (TIF) [file pone.0017669.s004.tif]

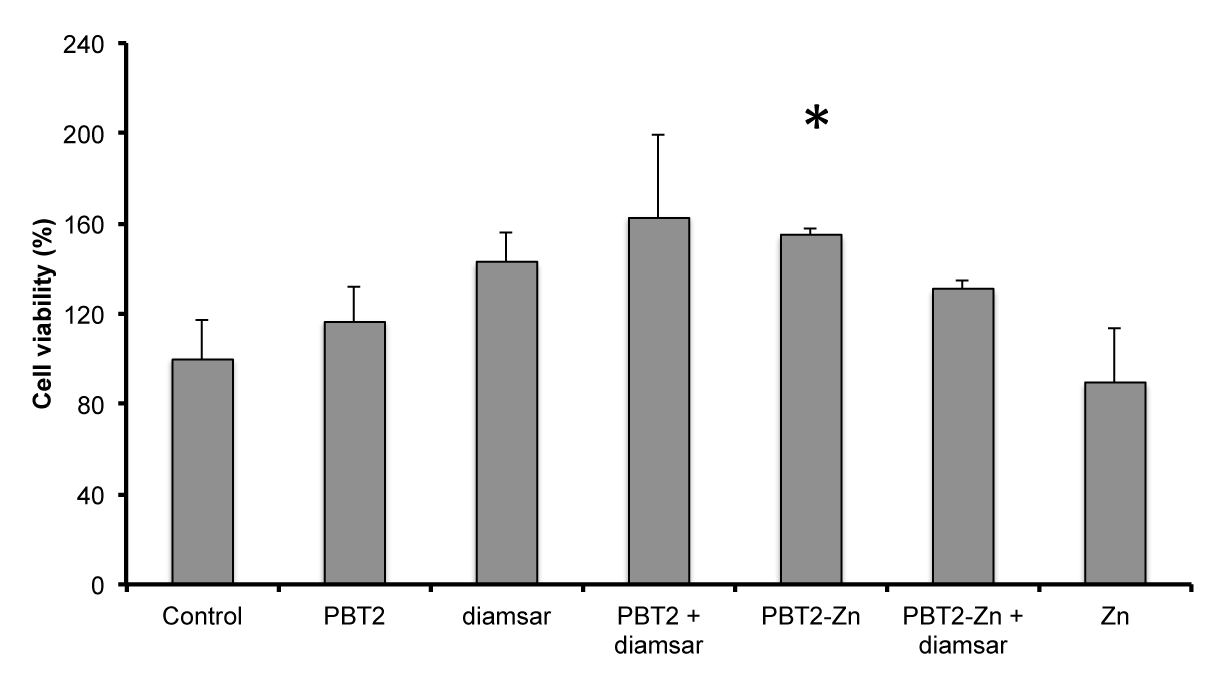

Supplement: Figure S5 — MTT assay on SH-SY5Y cells treated with PBT2 ± diamsar. PBT2-Zn treatment results in a significant increase in cell viability. Values are normalised to control = 100% and are means ± SEM *p<0.05. (TIF) [file pone.0017669.s005.tif]

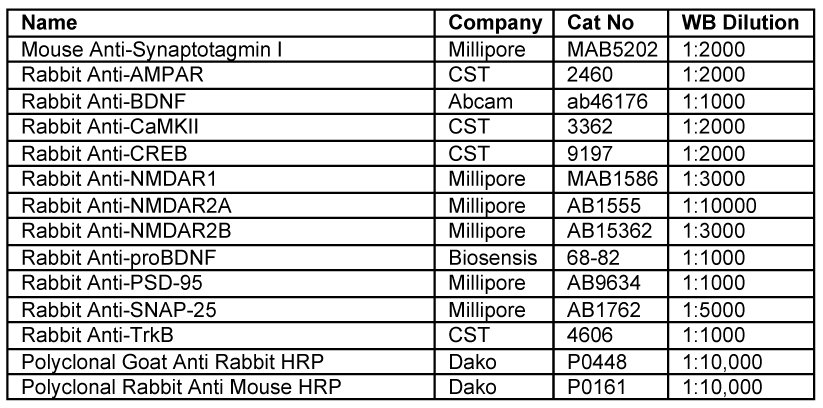

Supplement: Table S1 — The various antibodies used for western blot. (TIF) [file pone.0017669.s006.tif]
